# Demographic and Pathologic Characteristics of Malignant Melanoma in West Part of Romania

**Published:** 2018-04

**Authors:** Laura ENDRES, Diana UIVAROŞAN, Delia MIRELA ŢIŢ, Ovidiu POP, Simona BUNGĂU, Camelia BUHAŞ

**Affiliations:** 1. Dept. of Preclinical Disciplines, Faculty of Medicine and Pharmacy, University of Oradea, Oradea, Romania; 2. Dept. of Pharmacy, Faculty of Medicine and Pharmacy, University of Oradea, Oradea, Romania; 3. Dept. of Morphological Disciplines, Faculty of Medicine and Pharmacy, University of Oradea, Oradea, Romania

## Dear Editor-in-Chief

The consequence of the malignant transformation of melanocytes is cutaneous melanoma (MMC); the frequency of this disease is increasing especially in whites. The increased incidence in recent decades is due to the more intense exposure to the ultraviolet B radiation (UVR-B) (1), in addition to the genetic predisposition (2, 3). Despite some therapeutic advances, the prognosis of patients with MMC remains unfavorable. Median survival ranges are 6 to 8 months, with a 5 year survival rate of less than 5%. Metastatic melanoma remains incurable (4). The metastasis into regional lymph nodes is the most important prognostic factor in patients with early stages of melanoma and in 20% of the cases with intermediate-thickness melanoma (5, 6). In patients newly diagnosed with melanoma, biopsy of the sentinel node is suitable for cutaneous melanoma with intermediate thickness (between 1 and 4 mm Breslow index), with any anatomical location.

Our study was conducted in the Surgical Clinics of Clinical Emergency County Hospital Oradea and Pelican Hospital from Oradea, in collaboration with Oncology Institute Prof. Dr. Ion Chiricuţă from Cluj Napoca, for a period of 4 years (from 2010 to 2013), on a sample of 151 patients diagnosed with melanoma, in different stages of evolution.

The research was conducted in accordance with the WMA Declaration of Ethical Helsinki - Medical Research Involving Human Principles for Subjects. As well, it was approved by the Commission for internal approval of the research-development of the hospitals mentioned above and by the Ethics Commission of the Council of Faculty of Medicine and Pharmacy, University of Oradea, Oradea, Romania. Each patient included in the study signed an informed consent form.

As a result of surgical excision of the tumor formation and of the histopathological exam (7, 8), the cutaneous melanoma was diagnosed. It was located in various parts of the body, without any explanation: lip, eyelid, ear and external auditory canal, face, primary hairy skull and neck, trunk, upper limbs, lower limbs, lesions below the skin; in 52.31% of cases it was located at the level of the lips, trunk and lower limbs.

The 62 patients with cutaneous melanoma, with Breslow index between 1–4 mm, followed the biopsy procedure of the sentinel lymph node (SLN), in Oncology Institute form Cluj - Napoca. Overall, 45 patients had a positive SLN and 17 patients had negative SLN. The mortality rate was 5% in patients with positive SLN. By evaluating the death rates according to Breslow index, 65% of patients with Breslow index > 4 mm died. These results correspond with those cited in literature, the rate of survival in a Breslow index < 1 mm was between 95–100%, in a Breslow index 1–2 mm was 80–90%, in a Breslow index from 2.1 to 4 mm was 60–75%, and in a Breslow index > 4 mm is 50% (9).

We found by immunohistochemistry tests (S100, HMB45) the metastatic melanoma cells in subcapsular sinus as small cluster or large cluster. In some cases, the lymph node invasion was so important that the melanoma cells destroyed most of the normal lymphoide structure. We had a rare case of spindel cell melanoma.

Most patients are going to the doctor in an evolutionary stage of the disease, when there already exists a metastasized melanoma.
